# Microcystin-LR Induced Immunotoxicity in Mammals

**DOI:** 10.1155/2016/8048125

**Published:** 2016-01-26

**Authors:** Yaqoob Lone, Mangla Bhide, Raj Kumar Koiri

**Affiliations:** Department of Zoology, Dr. Harisingh Gour Central University, Sagar, Madhya Pradesh 470003, India

## Abstract

Microcystins are toxic molecules produced by cyanobacterial blooms due to water eutrophication. Exposure to microcystins is a global health problem because of its association with various other pathological effects and people all over the world are exposed to microcystins on a regular basis. Evidence shows that microcystin-LR (MC-LR) may adversely affect the immune system, but its specific effects on immune functions are lacking. In the present review, immunotoxicological effects associated with MC-LR in animals, humans, and* in vitro* models have been reported. Overall, the data shows that chronic exposure to MC-LR has the potential to impair vital immune responses which could lead to increased risk of various diseases including cancers. Studies in animal and* in vitro* models have provided some pivotal understanding into the potential mechanisms of MC-LR related immunotoxicity suggesting that further investigation, particularly in humans, is required to better understand the relationship between development of disease and the MC-LR exposure.

## 1. Introduction

**Figure 1:**
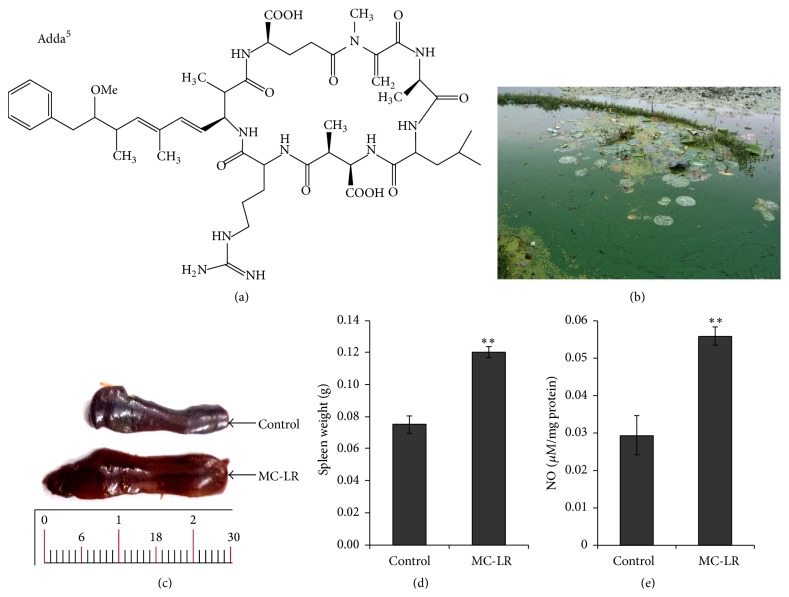
Structure of microcystin-LR (a) and* Microcystis aeruginosa* bloom in Sagar lake water (b) and MC-LR treatment of mice for 14 days shows splenomegaly (c) and causes a significant increase in the weight of spleen (d) and NO level in spleen (e). Values represent mean ± SD, where *n* = 3. ^*∗∗*^ *p* < 0.01 (control versus MC-LR treated mice).

The frequent occurrence of cyanobacterial blooms with increasing water eutrophication has become a worldwide concern. Cyanobacterial blooms are often coupled with the production of different ranges of bioactive and toxic metabolites with microcystins (MCs) being the most widely studied [1]. More than 90 microcystin isoforms have been detected, among which microcystin-leucine arginine (MC-LR, Figure 1(a)) is the most abundant and the most toxic variant of microcystin [2]. MC-LR is a potential carcinogen for animal and humans, and the International Agency for Research on Cancer has classified MC-LR as a possible human carcinogen due to its potential carcinogenic activity via inhibition of protein phosphatases, which leads to the hyperphosphorylation of cellular proteins [3]. The provisional guideline set by the World Health Organization for MC-LR in drinking water is 1 *μ*g/L, but the concentration of MCs in many water bodies is far beyond that guideline; for example, in Sagar lake water (India, Figure 1(b)) MC-LR was found to be 0.67 *μ*g/mL [4]. MC-LR, which is a well-known hepatotoxin, also induces damage in other organs as was supported by evidence of kidney impairment, gastrointestinal disorder, reproductive toxicity, immune intruders, and embryo toxicity [5–7]. There are reports which suggest that microcystin can alter the immune system through several mechanisms like lymphocyte proliferation reduction, modulation of phagocytic activity, adaptation of natural killer cell activity, and disturbance of cytokine synthesis [8, 9]. This lethal effect of MCs on human being and livestock considerably depends on the stimulation of their immune system; thus MCs alter the immunomodulatory activities [10]. In this review, an attempt has been made to figure out the information regarding conditions and mechanisms of immunotoxic activity of MCs.

## 2. Role of Hematological Parameters in Microcystin Mediated Immunotoxicity

Cyanobacteria produce a range of bioactive and toxic metabolites, which have been reported to bioaccumulate in aquatic food chains and have been reported to impact human health indirectly due to the presence of toxins in edible fish [11]. Various documented toxic effects of microcystins include chronic hepatocarcinogenicity and oxidative stress as well as modulations of hepatological parameters and immunosuppression via inhibition of IFN production and synthesis of cytokines [11]. Thus the immune system is prone to exposure and is sensitive to toxic agents. Previous studies have observed thrombocytopenia (platelet deficiency) in animals treated with MCs or cyanobacteria bloom extracts entirely containing MCs [12, 13]. Early investigations of mice treated with MCs have found thrombocytopenia, pulmonary thrombi, and hepatic congestion and it has been reported that rats treated with an acute dose of MC-LR (125 *μ*g/kg, i.p.) showed a significant decrease in WBC and mean corpuscular volume and a significant increase in platelets [12, 14]. Palikova et al. observed that mice fed with different concentration of cyanobacterial bloom extract for 28 days showed significant differences in RBC count, hematocrit value, MCH, MCV, and MCHC in comparison with the control group [11]. Previous results from the comet assay in mice leukocytes have shown that MC-LR (37.5 *μ*g/kg bw/day, i.p.) induced a 2-fold transient increase in the level of DNA breaks after 30 min exposure [15].* In vivo* studies by Kujbida et al. suggested that topical application of MC-LR (1000 nM) for 4 hours to male rats caused an improvement of the number of rolling and adhered leukocytes in the endothelium of postcapillary mesenteric venules [16].

Yuan et al. reported that, after the administration of MC-LR (50 *μ*g/kg bw), when blood was collected at 0, 1, and 3 hours and with 12 *μ*g/kg bw, when blood was collected at 0, 1, 3, 12, 24, 48, and 168 hours, respectively, significant increase in plasma white blood cells was observed [17]. Takahashi et al. reported that rats treated with MC-LR (100 and 200 *μ*g/kg, i.p.) for one hour showed dose dependent reductions in leukocyte count, erythrocyte count, hemoglobin (Hb) concentration, coagulation parameters, and hematocrit (Ht) [13]. Recently we investigated the effect of MC-LR (15 *μ*g/kg bw, i.p.) for 14 days on mice and observed that the treatment caused a significant elevation of hemoglobin and RBC, whereas it significantly declined WBC (Table 1, *p* < 0.005). Grabow et al. revealed that erythrocytes exposed to MC-LR had significant morphological changes [18]. Incubation of human erythrocytes with MC-LR concentrations of 1–1000 nM for 1, 6, 12, and 24 hours resulted in hemolysis and echinocytes and conversion of oxyhemoglobin to methemoglobin and a decrease in membrane fluidity [19]. Further in the treated erythrocytes activities of glutathione reductase and superoxide dismutase declined, while ROS and lipid peroxidation increased. Zhou et al. reported that when mice were exposed to MC-LR at the doses of 0.5, 2, and 8 *μ*g/kg bw every 48 h for 30 days, prominent decrease in RBC, Hb, and Ht was observed as compared to control [20].

## 3. Microcystins Activate Neutrophils and Macrophages

Neutrophils play significant role in regulation of cancer development and spontaneous tumorigenesis [21]. Activated neutrophils release ROS and play an important role in host defense system and removal of debris but they can also cause damage and injury to tissues [22]. Neutrophils have been reported to be involved in the liver injury induced by MC-LR [23]. Kujbida et al. had shown that MC-LR and [Asp^3^]-MC-LR increase migration of human neutrophils and ROS formation and its killing capacity [24]. Treatment of both rat and human neutrophils with MC-LA and MC-LR (1 and 1000 nM) for 24 hours has been observed to cause loss of membrane integrity as well an increase in percentage of cells with fragmented DNA in rats whereas in humans an increase in neutrophil viability and decrease in percentage of cells with fragmented DNA was observed [25]. Previous reports have suggested that MCs have a chemotactic effect [16, 26] and can attract neutrophil as well as enhance their migration, as the cells are induced to produce additional amounts of chemokine [16].* In vitro* studies have shown that all the three MCs cause neutrophil chemotaxis by increasing intracellular calcium levels [25].

Macrophages play an important role in immunity and foreign particles like microorganisms, macromolecules, and injured or apoptotic tissues [27] and other antigens are phagocytosed by macrophages [28]. Stimulated macrophages produce a number of enzymes, NO, and chemokines like IL-1*β*, TNF*α*, and GM-CSF for the primary protection of the host [29, 30]. In our studies, we have observed that concentration of NO in spleen increases significantly in mice treated with MC-LR (15 *μ*g/kg bw, i.p.) for 14 days (Figure 1(e)). Alterations in the level of cytokines or chemokines are considered as a marker of immunomodulation. Shen et al. examined the function of MCs on the phagocytosis of peritoneal cells in mice exposed to sublethal doses (16, 32, 64 mg/kg bw, i.p.) for 14 days and observed that MCs reduced phagocytic index of peritoneal phagocyte [31]. Microcytins also produced the inhibition of lipopolysaccharide induced lymphocyte proliferation and the dose dependent decrease of the numbers of antibody forming cells in mice that were immunized by using T-dependent antigen sheep red blood cells.* In vitro* studies of mice macrophages incubated with MC-LR at dose of 1, 10, 100, and 1000 nmol/L for 24 hours were studied by Chen et al. during which he observed the downregulation of NO production and mRNA levels of iNOS, IL-1*β*, and TNF*α* in peritoneal macrophages [32].

## 4. Microcystins Alter and Activate Lymphocyte

Using human and chicken peripheral blood lymphocytes treated with 1, 10, and 25 *μ*g/mL for 12, 24, 48, and 72 hours, Lankoff et al. showed that MC-LR influences the production of IL-2 and IL-6 and decreases the proliferation of T as well as B lymphocytes [33]. Human lymphocytes pretreated with MC-LR (1 *μ*g/mL) have been reported to increase the level of DNA damage as a function of time which might be due to apoptosis [33]. When mouse splenocytes were exposed to 7.5 *μ*g/mL of MC-LR for 4 and 24 hours, apoptosis was observed only in B cells whereas T cells were not affected [34]. We have also observed that treatment of mice with MC-LR (15 *μ*g/kg bw) for 14 days results in splenomegaly and significant increase in weight of spleen (Figures 1(c) and 1(d)) and cotreatment with nitrate was observed to potentiate this MC-LR induced toxicity in mice [35]. Flow cytometric analysis of nonstimulated lymphocytes treated with MC-LR (7.5 *μ*g/mL) for 4 and 24 hours has shown that MC-LR induces apoptosis in the B cell subpopulation via the B cell antigen receptor pathway, but not in T cells [34].

When mice were treated with three doses of MCs equivalent (4.97, 9.94, and 19.88 *μ*g/kg bw/i.p.) for 14 days, it was found that B cell was more susceptible, maybe due to the depression of B cell surface markers or B cell growth cytokines or their receptors by MCs [31]. Mice exposed at four doses of 7, 12, 24, and 36 mg/kg body weight for 8 hours resulted in a significant decrease of mRNA levels of TNF*α*, IL-1*β* (proinflammatory cytokines), and IL-4, IL-2, and IL-10 (Th1/Th2 related cytokines), while IL-6 level was unaffected [36]. In mice, prolonged exposure of MC-LR has been reported to cause DNA damage and inhibition of proliferation of bone marrow cells and changes of hematopoietic factors, which is an indicator of severe damage in bone marrow cells [20].

## 5. Conclusions

MC-LR is one of the most toxic cyanotoxins that has been extensively studied. However there are only few studies elucidating the possible connection between observed immunomodulating activities of MCs or other cyanotoxins. In the present review, an attempt has been made to comprehensively address the impact of MC-LR toxicity on immune system. In this paper, we have mainly described the* in vitro* and* in vivo* effect of MC-LR on both acute and chronic immune system of mice and an attempt has been made to describe the possible pathways and molecules which might be implicated in MC-LR induced immunotoxicity in mice.

## Figures and Tables

**Table 1:** Hematological parameters in blood of control and MC-LR treated mice for 14 days.

Hematological parameters	Control	Microcystin-LR
RBC (10^6^/mm^3^)	7.25 ± 0.72	3.12 ± 0.12^*∗∗*^
WBC (10^3^/mm^3^)	4.21 ± 0.29	5.73 ± 0.26^*∗∗*^
Hb (g/dL)	9.21 ± 0.25	6.23 ± 0.48^*∗∗*^

Values are mean ± SD (*n* = 3), ^*∗∗*^ *p* < 0.01 (control versus MC-LR treated groups).

## Abbreviations

GM-CSF: Granulocyte macrophage colony-stimulating factor
Hb: Hemoglobin
Ht: Hematocrit
IL: Interleukin
iNOS: Inducible nitric oxide synthase
MCH: Mean cell hemoglobin
MCHC: Mean corpuscular hemoglobin concentration
MC-LR: Microcystin-LR
MCs: Microcystins
MCV: Mean corpuscular volume
RBC: Red blood cells
ROS: Reactive oxygen species
TNF: Tumor necrosis factor
WBC: White blood cells.
